# Chlorophyll depletion and anthocyanin compositional shifts distinguish spotted leaf sectors in *Tricyrtis macropoda*

**DOI:** 10.3389/fpls.2026.1949134

**Published:** 2026-09-10

**Authors:** Yan Wang, Shanshan Han, Lisha Zhen, Yan Li, Fu Wang, Ying Wei

**Affiliations:** 1 Shaanxi Institute of Microbiology, Shaanxi Key Laboratory of Qinling Ecological Security, Shaanxi Academy of Sciences, Xi’an, Shaanxi, China; 2 Bio-Agriculture Institute of Shaanxi Province, Shaanxi Academy of Sciences, Xi’an, Shaanxi, China; 3 Institute of Botany of Shaanxi Province, Shaanxi Academy of Sciences, Xi’an, Shaanxi, China

**Keywords:** anthocyanin composition, chlorophyll depletion, gene–anthocyanin association, RNA-seq, spotted leaf, *Tricyrtis macropoda*

## Abstract

Sharply delimited leaf spots may reflect local changes in chlorophyll background, anthocyanin composition and gene expression, but these components are rarely resolved between adjacent green and spotted tissues. Three spatially defined leaf tissue types were sampled from one wild *T. macropoda* individual: green tissue from an upper non-spotted leaf (GGreen), and adjacent green (SGreen) and dark spotted (SSpot) tissues from the same lower spotted leaf. Three independently processed spatial subsamples were collected from each tissue type, yielding nine analytical samples. Chlorophyll content and targeted anthocyanin profiles were measured, and RNA sequencing was performed, followed by candidate transcript–anthocyanin association analysis and qRT–PCR of pooled samples. SSpot retained approximately half the chlorophyll measured in adjacent SGreen, whereas their summed abundance of detected anthocyanin derivatives did not differ significantly. Seven derivatives met the fold-change criterion in the SSpot–SGreen comparison, indicating a compositional shift rather than broad anthocyanin accumulation. RNA-seq separated all three tissue types; the dominant variation distinguished GGreen from both tissues of the spotted leaf, while the local SSpot–SGreen contrast contained 1,459 differentially expressed genes. An SGR-like chlorophyll-degradation candidate and transcripts assigned to anthocyanin biosynthesis, modification and transport showed SSpot-biased expression, and selected transcript abundances covaried with the seven fold-change-selected anthocyanins. The data define SSpot as a chlorophyll-depleted, compositionally distinct sector within the sampled individual and prioritize candidates for validation across plants and leaves.

## 1 Introduction

Patterned leaves often contain sharply delimited sectors that differ from the surrounding tissue in pigment composition, chloroplast status and gene expression. Whole-leaf sampling can obscure such local differences by averaging the tissue that defines the visible phenotype with the adjacent lamina (Bhardwaj et al., 2025). Studies of variegated leaves have linked colour variation to chloroplast development and pigment metabolism, most often through comparisons between green and discoloured regions, mutants or treatment groups (Zhang et al., 2019). Such designs are informative at the organ or genotype level, but they provide less resolution of local differences between adjacent colour sectors. Green tissue adjacent to a spot retains the same leaf context as the dark tissue, whereas green tissue from another leaf also differs in leaf identity, position and developmental history. In *Heliopsis helianthoides*, neighbouring sectors shared broad molecular features while retaining local differences in metabolite and transcript profiles (Qin et al., 2024), illustrating the value of resolving adjacent tissues before attributing a molecular signature to the visible sector itself. *T. macropoda* presents a comparable spatial problem because naturally occurring dark-brown spots are embedded within otherwise green lamina. Previous work in this species found differences in endophytic fungal communities and broad metabolite profiles between spotted and green leaf regions, with a higher relative abundance of *Cercospora* reported in the spotted regions (Wang et al., 2021). The biological basis of the dark-spotted phenotype, however, remains unresolved.

Visible colour does not necessarily track the abundance of a single pigment. Chlorophyll provides the green background, while anthocyanin colour depends on pigment concentration, molecular structure and the physicochemical environment within the vacuole (Tanaka et al., 2008). Vacuolar pH, metal complexation and co-pigmentation can alter anthocyanin hue and stability (Yoshida et al., 2009). Molecular transformations, self-association and acylation also contribute to anthocyanin colour expression (Hsu et al., 2024; Cao et al., 2024). Similar anthocyanin abundance can be associated with different visible colours when pigment composition or cellular conditions differ. Chlorophyll loss can alter this background independently of anthocyanin abundance. STAY-GREEN (SGR) proteins act as Mg-dechelatases during chlorophyll breakdown, and their contribution to chlorophyll loss has been characterized in both biochemical and physiological studies (Ren et al., 2023; Ando et al., 2024).

Anthocyanin composition reflects biosynthesis, structural modification and intracellular sequestration. The flavonoid pathway generates the core anthocyanidin structures (Winkel-Shirley, 2001), while downstream modification and vacuolar sequestration involve glycosylation, methylation and GST-, ABC- and MATE-associated systems (Pucker and Selmar, 2022; Zhao, 2015). Transcription factors, including MYB-family regulators, coordinate expression across the pathway (Li et al., 2022). Integrated transcriptome and metabolite profiling has identified pigment-associated pathways and candidate transcripts in a range of coloured-leaf systems (Zhang et al., 2022; Wu et al., 2024; He et al., 2025). Most studies, however, compare genotypes, developmental stages, treatments or whole-leaf states, leaving molecular variation between immediately adjacent green and coloured sectors less well resolved.

In this study, we characterized spatial variation in pigmentation and transcriptional profiles associated with the dark-spotted phenotype of *T. macropoda*. Three spatially defined tissue types were sampled from one wild individual: GGreen from an upper non-spotted leaf, and SGreen and SSpot from adjacent regions of the same lower spotted leaf. We measured chlorophyll content and anthocyanin profiles and performed RNA sequencing. Local differences were assessed primarily by comparing SSpot with adjacent SGreen. GGreen provided an additional comparison from a separate non-spotted leaf. Chloroplast- and anthocyanin-related transcripts identified in the local contrast were examined alongside the pigment measurements.

## 2 Materials and methods

### 2.1 Plant material and sector sampling

A wild individual of *T. macropoda* was collected from the northern slope of the Qinling Mountains, China (107°29′40″ E, 34°01′38″ N; 1,703 m above sea level), and identified by Professor Huyin Cheng (College of Pharmacy, Shaanxi University of Chinese Medicine, Xianyang, China). A voucher specimen was deposited in the Shaanxi Key Laboratory of Qinling Ecological Security under accession SQLES-2024-001. *T. macropoda* is not listed as endangered or vulnerable, and no specific permit was required for collection.

The intact plant was transported immediately to the laboratory. Leaves showing visible necrosis, insect damage or mechanical injury were excluded. All tissues were sampled from the same individual plant. GGreen was collected from a visually uniform, non-spotted leaf in the upper part of the plant. A lower leaf contained clearly defined dark-brown spots; green tissue immediately adjacent to selected spots was collected as SGreen, and the corresponding dark-spotted tissue was collected as SSpot. SGreen and SSpot therefore formed a within-leaf contrast, whereas GGreen provided a non-spotted reference from a different leaf of the same plant.

Three independently processed spatial subsamples were excised from each tissue type, yielding nine analytical samples. Each subsample was collected separately and processed independently for pigment measurements and RNA sequencing. These subsamples were used to characterize spatial variation within the sampled tissues and were not treated as independent plant- or leaf-level biological replicates. All tissues were frozen immediately in liquid nitrogen and stored at −80 °C until chlorophyll determination, targeted anthocyanin profiling, RNA sequencing and qRT-PCR analysis.

### 2.2 Chlorophyll determination

Leaf tissues stored at −80 °C were ground under liquid nitrogen for chlorophyll extraction. Approximately 0.08 g of the ground frozen tissue was extracted with 1 mL 95% ethanol and centrifuged at 6000 rpm for 2 min. The extraction was repeated three times, and the combined extract was adjusted to 10 mL with 95% ethanol. The extraction solvent was used as the blank.

Absorbance was measured at 663 and 646 nm using a 722N spectrophotometer. Chlorophyll a (Ca), chlorophyll b (Cb), and total chlorophyll (Ct), concentrations were calculated as follows (Ritchie, 2006):


$$\text{Ca}\;({\text{mg L}}^{-1})=12.72{\text{A}}_{663}-2.59{\text{A}}_{646}$$

$$\text{Cb}\;({\text{mg L}}^{-1})=22.88{\text{A}}_{646}-4.67{\text{A}}_{663}$$

$${\text{Ct}\;(\text{mg L}}^{-1})=\text{Ca}+\text{Cb}$$

Chlorophyll content was expressed on a fresh-weight basis and calculated as:

Chlorophyll content (mg g^−^¹ FW) = C × V × D/(1000 × W), where *C* is the chlorophyll concentration in the extract (mg L^−^¹), *V* is the final extract volume (mL), D is the dilution factor and *W* is the fresh mass of the tissue used for extraction (g).

### 2.3 Targeted anthocyanin profiling

Leaf samples were freeze-dried using a 100F freeze dryer (Scientz) and ground to powder using a MM400 ball mill (Retsch) at 30 Hz for 1.5 min. Targeted anthocyanins were extracted from 50 mg freeze-dried powder with 500 μL methanol/water (50:50, v/v) containing 0.1% hydrochloric acid. Samples were vortexed for 5 min using a MIX-200 vortex mixer (Shanghai Jingxin), sonicated for 5 min using a KQ5200E ultrasonic cleaner (Kunshan Shumei), and centrifuged at 12,000 × *g* for 3 min at 4 °C using a 5424R centrifuge (Eppendorf). The residue was extracted once more under the same conditions. Combined supernatants were filtered through a 0.22 μm membrane before LC–MS/MS analysis.

Anthocyanins were quantified using an ExionLC™ AD UPLC system coupled to a QTRAP^®^ 6500+ LC–MS/MS system (SCIEX). Chromatographic separation used an ACQUITY UPLC BEH C18 column (2.1 mm × 100 mm, 1.7 μm) at 40 °C. Mobile phase A was water with 0.5% formic acid, and mobile phase B was methanol with 0.5% formic acid. The gradient was 5% B at 0 min, 50% B at 6 min, 95% B at 12 min, held for 2 min, returned to 5% B at 14 min and re-equilibrated for 2 min. The flow rate was 0.35 mL/min, and the injection volume was 2 μL.

The mass spectrometer was operated in positive electrospray ionization mode with a Turbo Ion-Spray interface. The source temperature was 550 °C, the ion spray voltage was 5500 V and the curtain gas was 35 psi. Anthocyanins were detected in scheduled multiple reaction monitoring mode. Declustering potential and collision energy were optimized for each MRM transition.

Anthocyanin identification was based on retention time, precursor/product ion transitions and comparison with a standard-based MetWare database. Quantification was performed from integrated MRM peak areas using external calibration curves generated from serial standard solutions. Standard solutions ranged from 0.01 to 5000 ng/mL, and compound concentrations were converted to μg/g dry sample according to the sample mass and extraction volume. Mass-spectrometric data were acquired with Analyst 1.6.3 software and processed with MultiQuant 3.0.3 software. Compounds not quantified in individual samples were recorded as not detected in the sample-level matrix. For abundance summaries, non-detected values were entered as zero according to the quantitative output matrix.

Pooled quality-control samples were used to monitor analytical stability during LC–MS/MS acquisition. Instrument performance and data reproducibility were assessed from total ion chromatogram overlap and the coefficient of variation distribution of QC samples.

Total detected anthocyanin derivatives were calculated as the summed abundance of quantified anthocyanin derivatives in each sample. For class-level summaries, quantified anthocyanin derivatives were grouped according to their aglycone class, including cyanidin, delphinidin, petunidin, malvidin, pelargonidin and peonidin derivatives. Anthocyanin derivatives were screened among tissue types using fold change ≥ 2 or ≤ 0.5 based on group-level quantitative values.

### 2.4 RNA sequencing, assembly and annotation

Total RNA was isolated separately from each independently processed spatial subsample using CTAB–PBIOZOL extraction followed by ethanol precipitation and was dissolved in 50 μL of DEPC-treated water. RNA concentration was measured with a Qubit 4.0 fluorometer, and integrity was assessed with a Qsep400 Bio-fragment Analyzer. RNA samples that met the quality requirements were used for library preparation.

Poly(A)-tailed RNA was captured with oligo(dT) magnetic beads and fragmented. First-strand cDNA was synthesized with random hexamer primers, followed by second-strand synthesis. Double-stranded cDNA was purified, end-repaired, A-tailed, ligated to sequencing adaptors, size-selected and amplified by PCR.

The libraries were sequenced on an Illumina platform. Sequencing of the nine independently processed spatial subsamples yielded 64.72 Gb of clean data. Each library contributed at least 6 Gb, and Q30 values were ≥92%. Raw reads were processed with fastp v0.23.2 (Chen et al., 2018). Reads containing adaptor sequences were removed, as were read pairs in which either mate contained more than 10% ambiguous bases or more than 50% bases with a quality score of Q ≤ 20. Clean reads were assembled *de novo* using Trinity v2.15.1 (Haas et al., 2013). Transcripts were clustered with Corset v1.09 (Davidson and Oshlack, 2014), and the longest sequence in each cluster was retained as the representative unigene. The final assembly contained 91,097 transcripts with an N50 of 1,515 bp and 46,965 unigenes with an N50 of 1,611 bp. Coding regions were predicted with TransDecoder v5.3.0.

Functional annotation was performed by searching the unigene sequences against the KEGG (Kanehisa et al., 2023), NR, Swiss-Prot, GO, COG/KOG and TrEMBL databases (Bateman et al., 2023) with DIAMOND v2.0.9 (Buchfink et al., 2021). Protein domains were identified against Pfam (Mistry et al., 2021) using HMMER (Finn et al., 2011). Of the 46,965 unigenes, 33,406 (71.13%) received at least one database annotation. Transcription-factor families were assigned using iTAK (Zheng et al., 2016).

Clean reads from each spatial subsample were mapped back to the assembled transcriptome with Bowtie2 v2.4.5 (Langmead and Salzberg, 2012) as implemented in RSEM v1.3.1 (Li and Dewey, 2011). Mapping rates ranged from 88.87% to 89.53%. RSEM-derived fragments per kilobase of transcript per million mapped fragments (FPKM) values were used for expression visualization and descriptive analyses, whereas the corresponding raw count matrix was retained for differential-expression testing.

### 2.5 Differential expression, DEG-set analysis and functional enrichment

Raw read counts were analysed with DESeq2 v1.22.2 (Love et al., 2014) for three pairwise contrasts: SGreen versus GGreen, SSpot versus GGreen and SSpot versus SGreen. P values were adjusted using the Benjamini–Hochberg procedure (Benjamini and Hochberg, 1995). Transcripts with |log_2_ fold change| ≥ 1 and FDR < 0.05 were classified as differentially expressed genes (DEGs). Positive log_2_ fold-change values indicate higher expression in the first-named tissue.

Mutually exclusive intersections were generated by matching transcript identifiers across the complete DESeq2 result tables. Genes meeting the DEG criteria in the SSpot–SGreen contrast, but not in either GGreen-referenced contrast, were assigned to the SSpot–SGreen-exclusive intersection. Genes shared by the SGreen–GGreen and SSpot–GGreen contrasts were classified as direction-consistent when their log_2_ fold changes had the same sign in both comparisons.

For the expression heatmap of the SSpot–SGreen-exclusive intersection, FPKM values were transformed as log_2_(FPKM + 1) and standardized across samples for each gene. Genes with higher and lower expression in SSpot were clustered separately using Euclidean distance and complete linkage. Columns were retained in the order GGreen, SGreen and SSpot, with three independently processed spatial subsamples for each tissue type.

GO and KEGG over-representation analyses were performed for the complete SSpot–SGreen DEG set and for the direction-consistent genes shared by the two GGreen-referenced contrasts. The tested universe contained 21,321 transcripts with valid differential-expression statistics in all three contrasts. The GO and KEGG backgrounds comprised 17,187 and 6,846 annotated genes, respectively. Enrichment was tested using one-sided hypergeometric tests, followed by Benjamini–Hochberg correction (Benjamini and Hochberg, 1995) separately for each gene set and annotation database. Terms represented by fewer than two genes from the input set were omitted. KEGG pathways with FDR < 0.05 in at least one gene set were retained for the main figure. Complete GO and KEGG over-representation results are provided in **Supplementary Tables 4**, **5**, respectively.

### 2.6 Gene–anthocyanin association analysis

Transcript abundance and targeted anthocyanin concentrations were integrated across the same nine independently processed spatial subsamples. The analysis included 10 representative TF-family candidates, 12 transcripts annotated in anthocyanin biosynthesis, modification or transport, and the seven fold-change-selected anthocyanins that differed between SSpot and SGreen. Transcript abundance was transformed as log_2_(FPKM + 1). Anthocyanin abundance was log_2_-transformed after addition of a compound-specific pseudocount equal to one-half of the smallest positive value recorded for that compound.

Pearson’s correlation coefficient was calculated in R v4.5.1 for all 154 transcript–anthocyanin pairs. P values were adjusted across all tested pairs using the Benjamini–Hochberg procedure (Benjamini and Hochberg, 1995). Associations with ∣r∣ ≥ 0.80 and FDR < 0.05 were displayed in the association matrix. No filtering based on the direction of the SSpot–SGreen anthocyanin difference was applied. The analysis describes covariation across the sampled sectors and was not used to infer direct regulatory relationships.

### 2.7 qRT-PCR

Fifteen transcription-factor-family targets were examined by qRT–PCR. One pooled sample was analyzed for each tissue type (GGreen, SGreen and SSpot). The pooled qRT–PCR measurements were used for descriptive comparison with the corresponding RNA-seq expression patterns and were not treated as independent biological replicates. Primer sequences, amplicon sizes, assay-to-transcript correspondence, pooled expression summaries and RNA-seq comparison results are provided in **Supplementary Table 1**.

First-strand cDNA was synthesized from approximately 2 μg of total RNA from each pooled sample using a reverse-transcription system with dsDNase treatment. Each 10-μL PCR reaction contained 1 μL cDNA, 0.7 μL forward primer, 0.7 μL reverse primer, 5 μL 2× SYBR Green PCR Master Mix, 0.05 μL QN ROX Reference Dye and 2.55 μL RNase-free water. Amplification was performed on an ABI 7500 Real-Time PCR System (Applied Biosystems, Thermo Fisher Scientific) at 95 °C for 2 min, followed by 40 cycles of 95 °C for 5 s and 60 °C for 30 s and subsequent melting-curve analysis.

Each pooled sample was analysed in three technical PCR reactions. Relative transcript abundance was calculated using the 2^−ΔΔCt^ method (Livak and Schmittgen, 2001), with *atp1* as the reference gene and the GGreen pool as the calibrator. Technical-reaction-level Ct, ΔCt, ΔΔCt and relative-expression values are provided in **Supplementary Data 3**.

### 2.8 Statistical analysis and visualization

Pigment measurements are presented as mean ± s.d. from three independently processed spatial subsamples per tissue type. Differences among GGreen, SGreen and SSpot were tested by one-way analysis of variance followed by Tukey’s honestly significant difference test in IBM SPSS Statistics 26.0. Statistical significance was defined as *P* < 0.05. Statistical comparisons were restricted to within-individual spatial variation.

qRT–PCR data are shown as mean ± s.d. across three technical reactions for each pooled sample. No inferential statistical tests were applied. Directional concordance with RNA sequencing was defined by agreement in the sign of the SSpot–SGreen change for assays present in the corresponding DESeq2 output.

Transcriptome ordination, sample-correlation analysis, DEG-set reconstruction, enrichment testing and figure preparation were conducted in R 4.5.1. Transcripts with zero FPKM in all nine samples were removed before exploratory expression analysis. Principal component analysis was performed on centred, unscaled log_2_(FPKM + 1) values for the 5,000 genes with the highest variance across the nine samples. Sample similarity was calculated using Pearson correlations across all genes expressed in at least one sample, using the same transformed expression values.

**Figure 1:**
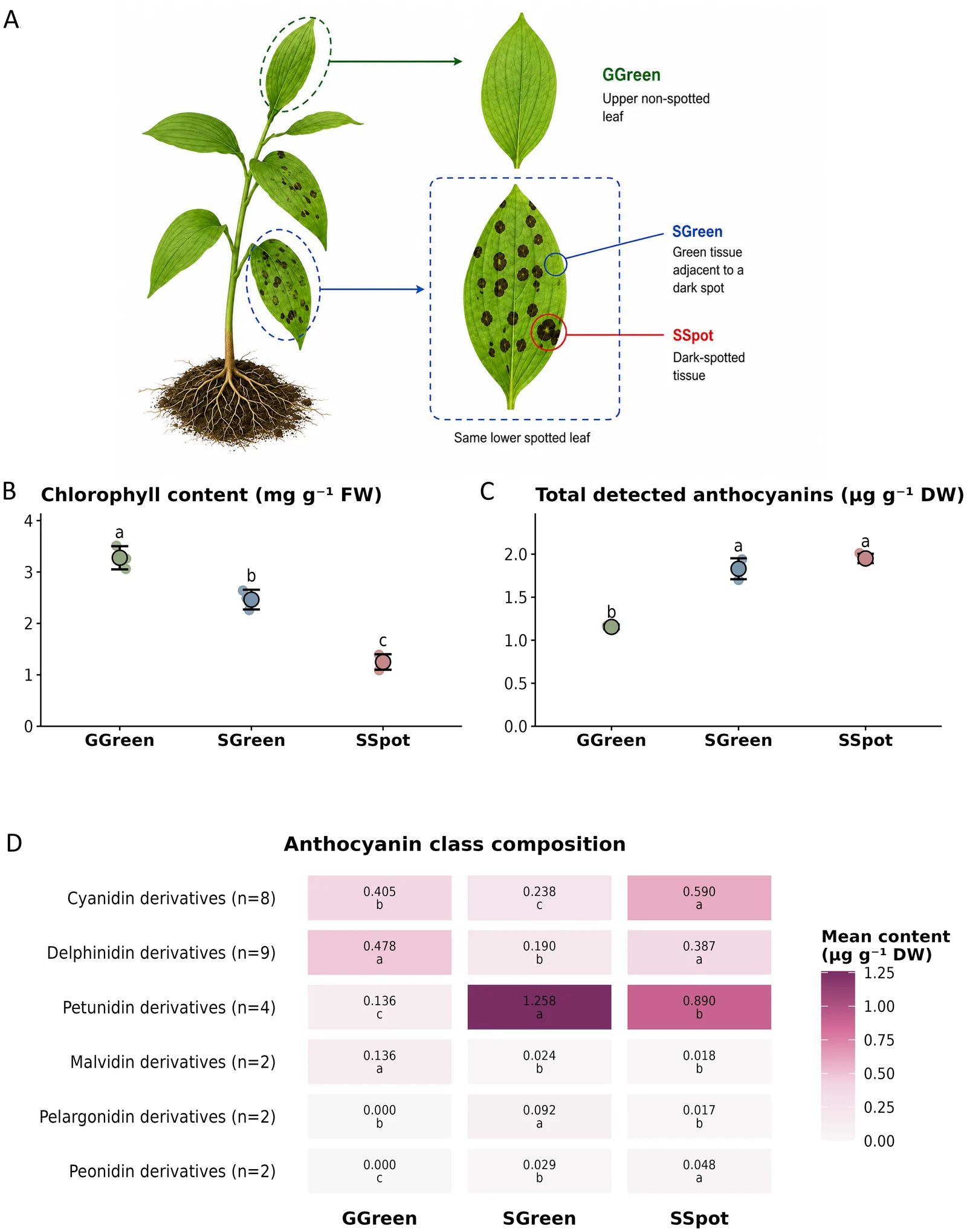
Sampling scheme and pigment variation among leaf tissues of *Tricyrtis macropoda*. **(A)** Sampling scheme. GGreen was collected from an upper non-spotted leaf. SGreen and SSpot were collected from adjacent green and dark-spotted regions of the same lower spotted leaf. **(B)** Chlorophyll content. **(C)** Total content of all 27 detected anthocyanin derivatives. **(D)** Mean content of the 27 detected anthocyanin derivatives grouped by aglycone class. In B and C, small points show the three independently processed spatial subsamples, and larger points with error bars show the mean ± s.d. Different lowercase letters indicate significant differences among tissue types (*P* < 0.05, one-way ANOVA followed by Tukey’s honestly significant difference test). In D, values are mean contents for each anthocyanin class, and different lowercase letters indicate significant differences among tissue types within that class using the same test. The number of detected derivatives assigned to each class is given in parentheses. FW, fresh weight; DW, dry weight.

Heatmaps were based on gene-wise standardized expression values. For the DEG heatmap in **Figure 1**, the 100 genes with the highest variance among the 6,158 genes detected as differentially expressed in at least one contrast were selected and ordered by complete-linkage hierarchical clustering. Sample columns were displayed in the fixed order GGreen, SGreen and SSpot.

Data import and processing used readxl 1.5.0, readr 2.1.5, dplyr 1.1.4, tidyr 1.3.1 and stringr 1.5.1. Graphics were prepared with ggplot2 3.5.2, ComplexHeatmap 2.24.1, circlize 0.4.16, patchwork 1.3.1 and scales 1.4.0.

## 3 Results

### 3.1 Spotted tissue shows chlorophyll depletion and altered anthocyanin abundance patterns

**Figure 2:**
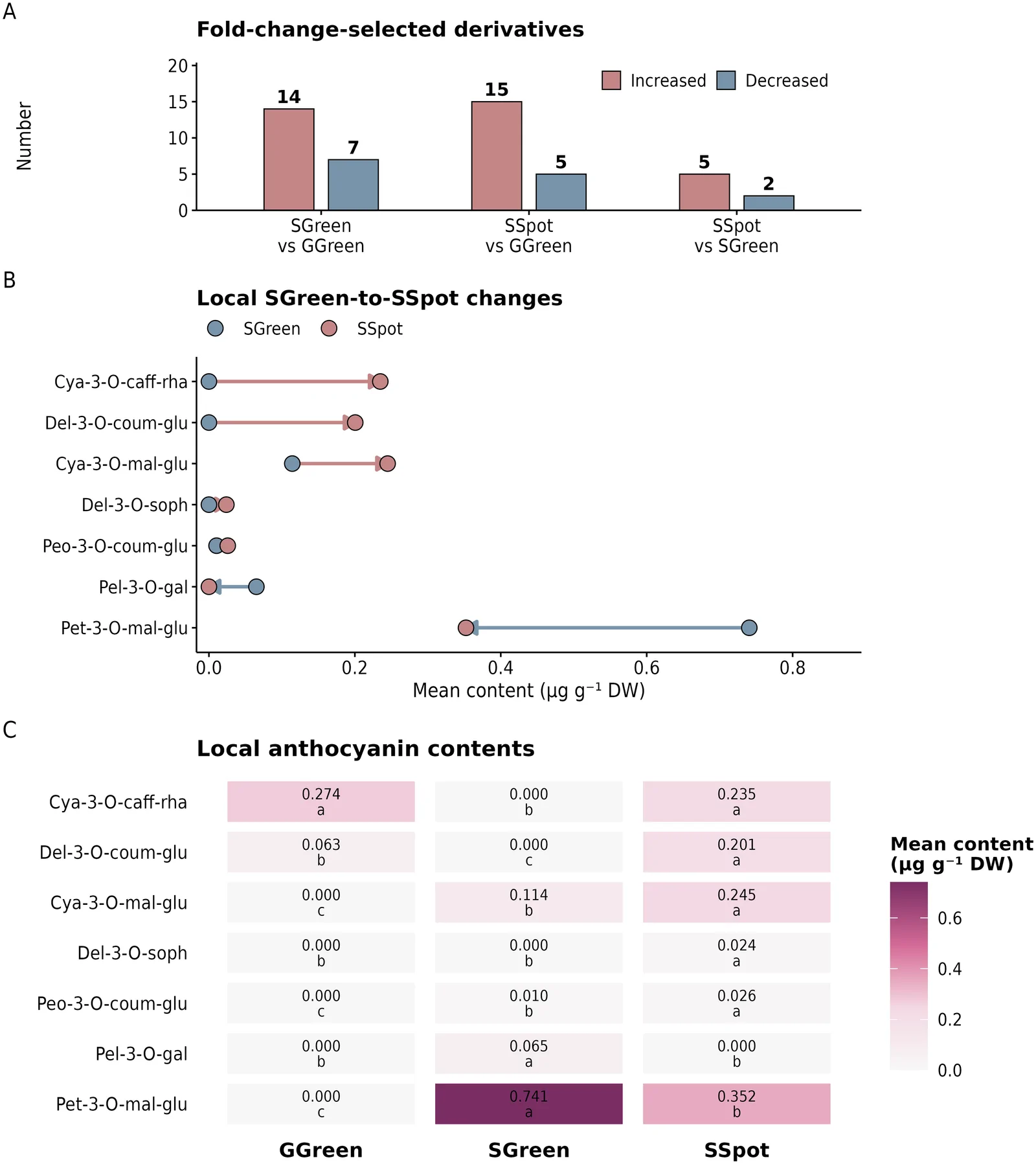
Fold-change-selected anthocyanin derivatives across GGreen, SGreen and SSpot. **(A)** Numbers of anthocyanin derivatives meeting the fold-change threshold in each pairwise comparison. Increased and decreased abundance are defined relative to the first-named tissue in each comparison. **(B)** Changes in mean content of the seven anthocyanin derivatives meeting the fold-change criterion in the SSpot–SGreen comparison. **(C)** Mean contents of the same seven fold-change-selected anthocyanin derivatives across GGreen, SGreen and SSpot. Derivatives were retained using a fold-change threshold of ≥2 or ≤0.5 based on group mean abundance. Contents are expressed as μg g^−^¹ DW. Values in C are group means calculated from the three independently processed spatial subsamples per tissue type. Different lowercase letters denote *P* < 0.05 by one-way ANOVA followed by Tukey’s honestly significant difference test. Cya, cyanidin derivative; Del, delphinidin derivative; Peo, peonidin derivative; Pel, pelargonidin derivative; Pet, petunidin derivative; mal, malonyl; caff, caffeoyl; coum, coumaroyl; soph, sophoroside; glu, glucoside; rha, rhamnoside; gal, galactoside.

Three tissue types from the same individual of *T. macropoda* were compared. GGreen was sampled from a visually uniform, non-spotted leaf in the upper part of the plant. SGreen and SSpot were collected from a lower spotted leaf and represented green tissue bordering a dark spot and the corresponding dark tissue, respectively (**Figure 2A**). Thus, SGreen and SSpot formed a within-leaf contrast, whereas GGreen provided a non-spotted reference from another leaf of the same plant.

Total chlorophyll differed markedly among the three tissue types. GGreen contained the highest concentration (3.28 ± 0.23 mg g^−^¹ FW), followed by SGreen (2.46 ± 0.19 mg g^−^¹ FW), whereas SSpot contained the lowest concentration (1.25 ± 0.15 mg g^−^¹ FW; **Figure 2B**). Chlorophyll in SSpot corresponded to approximately 38% of that measured in GGreen and 51% of that measured in adjacent SGreen.

The summed abundance of detected anthocyanin derivatives showed a different pattern. Both SGreen and SSpot contained more detected anthocyanins than GGreen, with mean values of 1.154, 1.831 and 1.950 μg g^−^¹ dry weight for GGreen, SGreen and SSpot, respectively (**Figure 2C**). SGreen and SSpot did not differ significantly in total detected anthocyanin abundance. The dark SSpot phenotype was therefore accompanied by pronounced chlorophyll depletion, but only a modest change in the summed anthocyanin pool relative to adjacent SGreen.

Anthocyanin class composition further separated SSpot from the two green tissues (**Figure 2D**). SSpot contained the highest summed abundance of cyanidin and peonidin derivatives. Petunidin derivatives were most abundant in SGreen, which also contained most of the detected pelargonidin derivatives. GGreen contained the highest abundance of malvidin derivatives, whereas delphinidin derivatives were less abundant in SGreen than in either GGreen or SSpot. Thus, the local SGreen–SSpot contrast was characterized more clearly by a redistribution among detected anthocyanin classes than by a broad increase in total anthocyanin abundance.

### 3.2 SSpot differs from adjacent SGreen by a focused shift in anthocyanin composition

**Figure 3:**
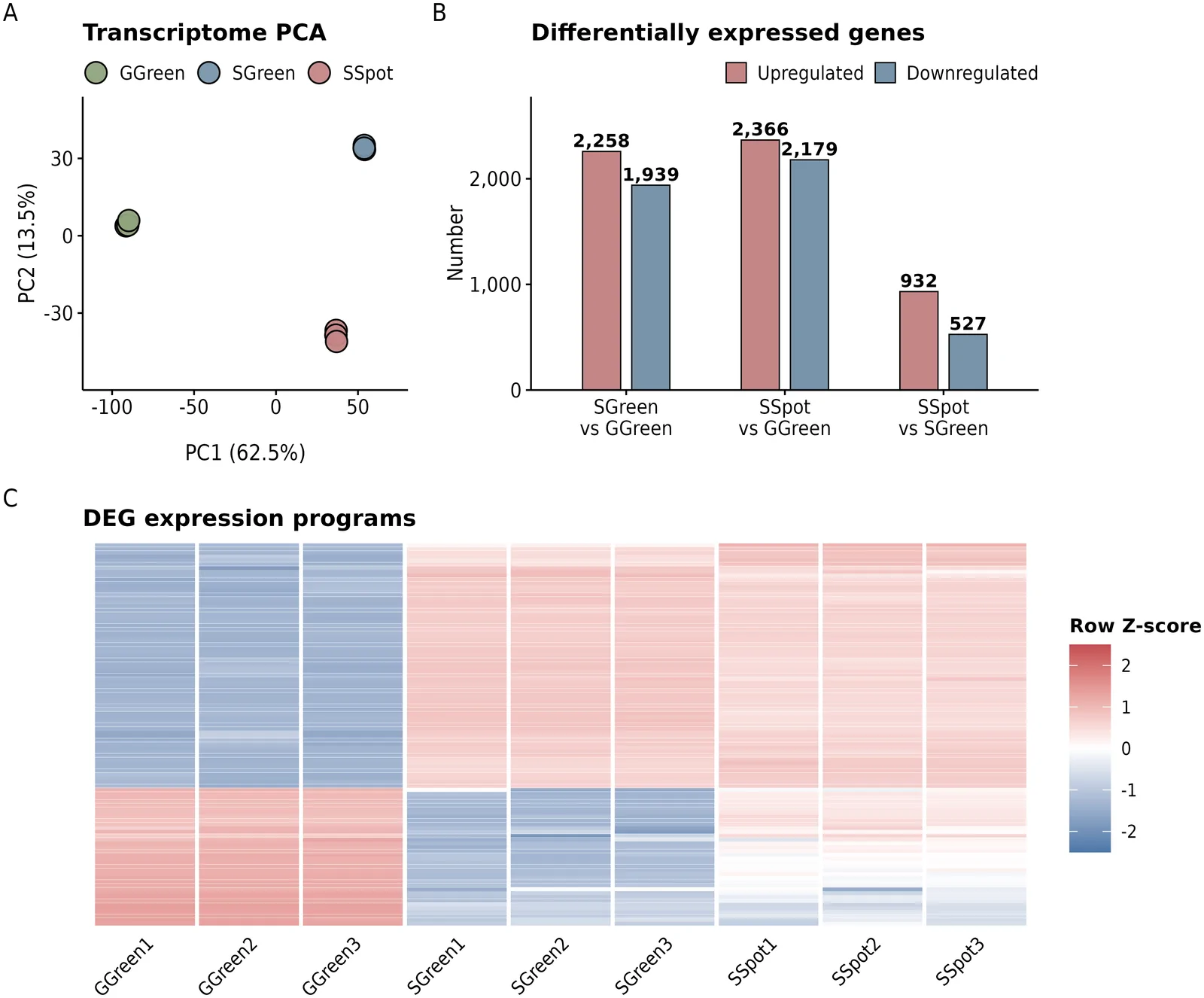
Transcriptome profiles of GGreen, SGreen and SSpot tissues. **(A)** Principal component analysis based on log_2_(FPKM + 1)-transformed expression values for the 5,000 genes with the greatest variance across the nine independently processed spatial subsamples. Each point represents one spatial subsample. **(B)** Numbers of genes with higher or lower expression in the three pairwise comparisons. Differentially expressed genes were identified from raw read counts using DESeq2 and defined by |log_2_ fold change| ≥ 1 and FDR < 0.05. Higher and lower expression are defined relative to the first-named tissue in each comparison. **(C)** Heatmap of selected differentially expressed genes across the nine spatial subsamples. Values are row-scaled log_2_(FPKM + 1) expression levels.

Anthocyanin derivatives were screened using the prespecified fold-change threshold of ≥2 or ≤0.5. Relative to GGreen, 23 derivatives met this criterion in SGreen, of which 14 were more abundant and 9 less abundant. The corresponding comparison between SSpot and GGreen retained 22 derivatives, including 15 with higher and 7 with lower abundance in SSpot (**Figure 3A**). The within-leaf SSpot–SGreen comparison identified seven derivatives meeting the same criterion: five were more abundant in SSpot and two were less abundant. The complete abundance profiles and class assignments of all 27 detected anthocyanin derivatives are provided in **Supplementary Table 2**.

The five derivatives with higher abundance in SSpot were Cyanidin-3-O-(6′′-O-caffeoyl)rhamnoside, Cyanidin-3-O-(6-O-malonyl-*β*-D-glucoside), Delphinidin-3-O-(6-O-*p*-coumaroyl)-glucoside, Delphinidin-3-O-sophoroside and Peonidin-3-O-(6-O-*p*-coumaroyl)-glucoside (**Figures 3B, C**). Pelargonidin-3-O-galactoside and Petunidin-3-O-(6-O-malonyl-*β*-D-glucoside) were less abundant in SSpot than in SGreen. The latter remained one of the most abundant derivatives in both tissues, but its mean concentration declined from 0.743 μg g^−^¹ in SGreen to 0.352 μg g^−^¹ in SSpot. By contrast, several cyanidin- and delphinidin-derived compounds increased in SSpot, including Cyanidin-3-O-(6-O-malonyl-*β*-D-glucoside), which rose from 0.114 to 0.245 μg g^−^¹, and Delphinidin-3-O-(6-O-*p*-coumaroyl)-glucoside, which was not detected in SGreen but reached 0.201 μg g^−^¹ in SSpot. These changes indicate that the local SGreen–SSpot contrast was concentrated in a small number of specific derivatives rather than in a uniform increase across the detected anthocyanin pool.

### 3.3 Transcriptome profiling separates the three leaf tissue types

RNA-sequencing profiles differed across the nine independently processed spatial subsamples. In principal component analysis of the 5,000 most variable expressed genes, GGreen, SGreen and SSpot occupied distinct regions of the score plot (**Figure 1A**). PC1 accounted for 62.5% of the total variance and separated GGreen from the two tissues sampled from the lower spotted leaf. PC2 accounted for a further 13.5% and distinguished SGreen from SSpot. A Pearson correlation matrix for the nine spatial subsamples is provided in **Supplementary Figure 1**.

Differential-expression analysis was performed on RSEM-derived raw counts using DESeq2, with |log_2_ fold change| ≥ 1 and FDR < 0.05. The SGreen–GGreen comparison identified 4,197 differentially expressed genes, comprising 2,258 genes with higher expression in SGreen and 1,939 with lower expression. The SSpot–GGreen comparison identified 4,545 genes, of which 2,366 were higher and 2,179 lower in SSpot. The within-leaf SSpot–SGreen comparison yielded 1,459 differentially expressed genes, including 932 with higher and 527 with lower expression in SSpot (**Figure 1B**). Both comparisons involving GGreen contained substantially more DEGs than the SSpot–SGreen comparison.

The expression heatmap revealed two broad patterns across the sampled tissues (**Figure 1C**). One gene set showed lower expression in GGreen and higher expression in both SGreen and SSpot, whereas a second set showed the opposite pattern. SGreen and SSpot also differed for a subset of genes, consistent with their separation along PC2.

### 3.4 Shared and local transcriptional patterns distinguish leaf-context and within-leaf contrasts

**Figure 4:**
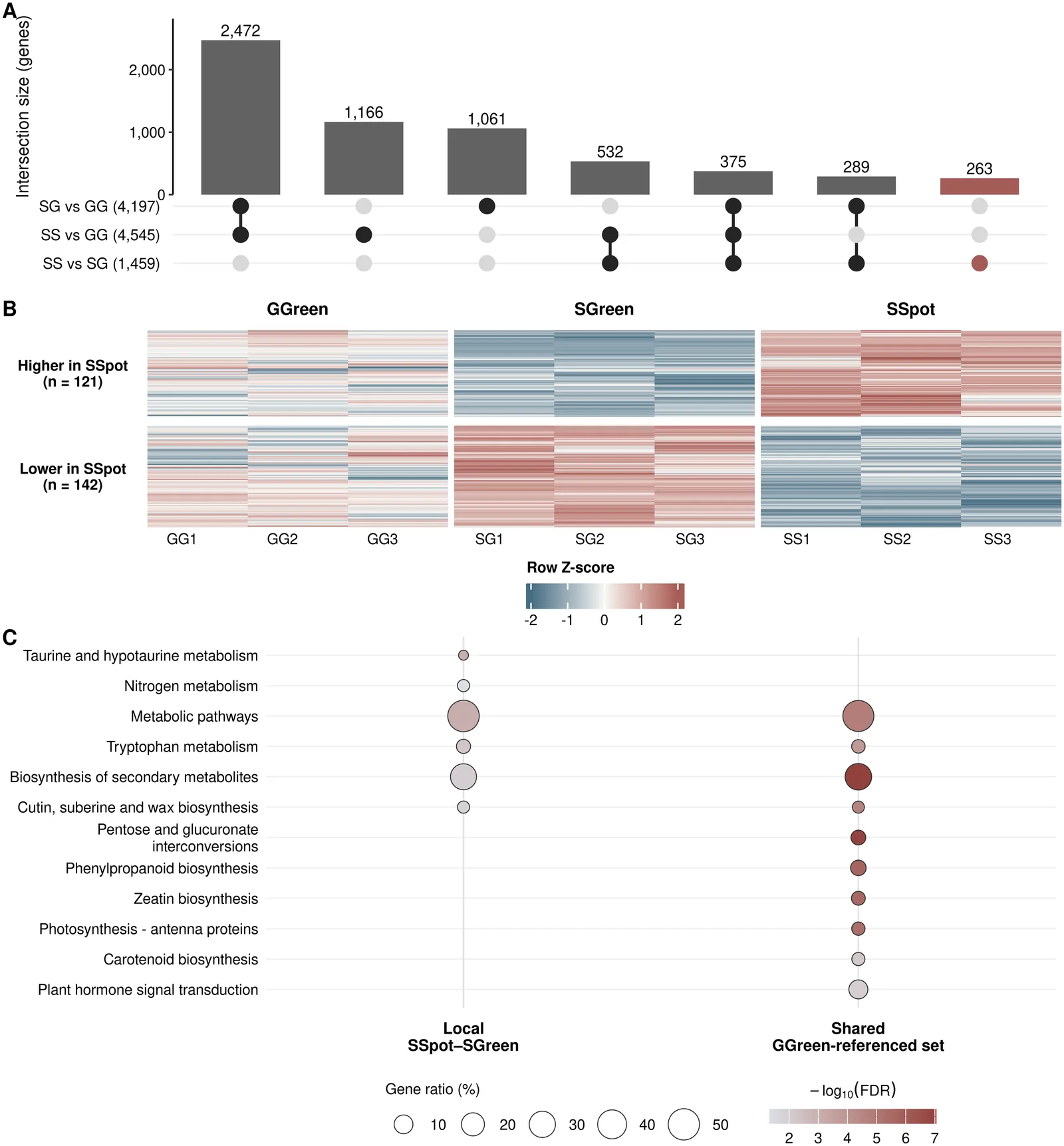
Shared and local transcriptional differences across the sampled tissues. **(A)** UpSet plot of DEGs identified in the SGreen–GGreen, SSpot–GGreen and SSpot–SGreen comparisons using ∣log_2_FC∣ ≥ 1 and FDR < 0.05. Bars show mutually exclusive intersections; the red intersection denotes genes meeting the DEG criteria only in the SSpot–SGreen comparison. **(B)** Row-scaled log_2_(FPKM + 1) expression of these 263 genes across nine independently processed spatial subsamples. Genes are grouped by the direction of the SSpot–SGreen difference and clustered within each group. **(C)** KEGG enrichment of the 1,459 SSpot–SGreen DEGs and the 2,838 same-direction genes shared by the two GGreen-referenced comparisons. Dot size indicates gene ratio and colour indicates −log10(FDR). Only pathways with FDR < 0.05 in at least one gene set are shown. SG, SGreen; SS, SSpot; GG, GGreen.

A total of 6,158 transcripts met the differential-expression criteria in at least one of the three contrasts. The largest mutually exclusive intersection contained 2,472 genes detected in both GGreen-referenced comparisons but not in SSpot–SGreen, whereas 375 genes were differentially expressed in all three contrasts (**Figure 4A**). The SGreen–GGreen and SSpot–GGreen comparisons shared 2,847 DEGs, of which 2,838 changed in the same direction. A separate set of 263 genes met the thresholds only in the SSpot–SGreen comparison; 121 were higher and 142 were lower in SSpot (**Figure 4B**). Detailed DEG counts and mutually exclusive intersections are provided in **Supplementary Table 3**. The complete 6,158-gene DEG union and the expression matrix for the 263-gene SSpot–SGreen-exclusive set are provided in **Supplementary Data 1**, **2**, respectively.

The 1,459 SSpot–SGreen DEGs were enriched for pathways associated with primary and secondary metabolism, including tryptophan, nitrogen, carotenoid and flavonoid metabolism, as well as taurine and hypotaurine metabolism and cutin, suberine and wax biosynthesis (**Figure 4C**; **Supplementary Table 5**). GO over-representation analysis of this set identified flavonoid biosynthetic and metabolic processes, tetrapyrrole binding, phenylpropanoid biosynthesis and chalcone synthase activity among the significantly enriched terms (**Supplementary Table 4**). The 2,838 direction-consistent transcripts shared by the two GGreen-referenced comparisons were enriched for pentose and glucuronate interconversions, phenylpropanoid biosynthesis, carotenoid biosynthesis, photosynthesis–antenna proteins, zeatin biosynthesis and plant hormone signal transduction. KEGG flavonoid biosynthesis contained seven SSpot–SGreen DEGs and met the applied multiple-testing threshold (P = 0.0040, FDR = 0.0445) (**Supplementary Table 5**).

### 3.5 Local expression changes in chlorophyll- and photosynthesis-associated transcripts

**Figure 5:**
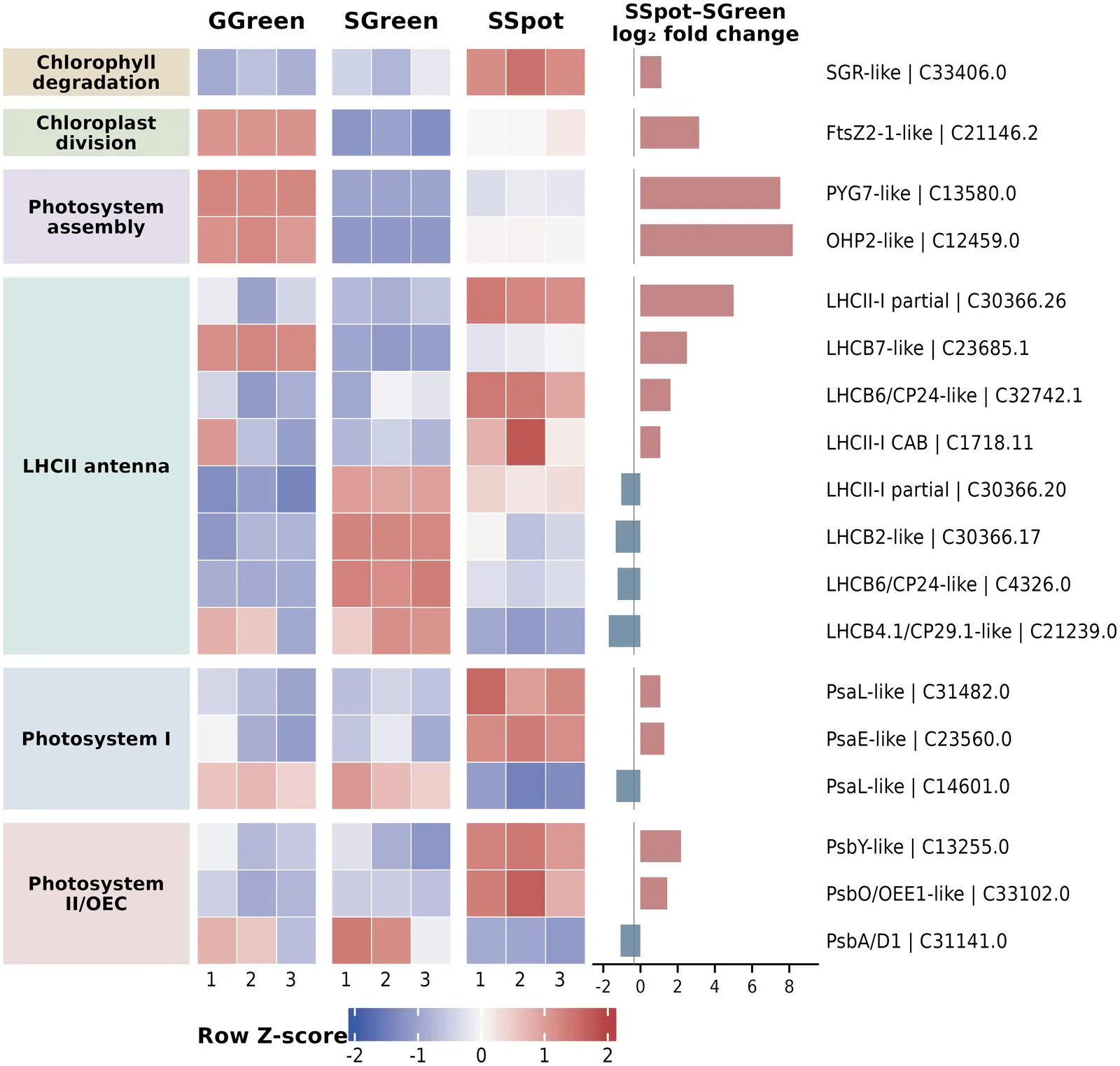
Chlorophyll- and photosynthesis-associated transcripts in the local SSpot–SGreen contrast. Expression profiles of 18 sequence-supported transcripts across three independently processed spatial subsamples of GGreen, SGreen and SSpot. Heatmap colours represent row-standardized log_2_(FPKM + 1) values and relative expression across samples within each transcript. Transcripts are grouped by curated functional annotation. Bars indicate the DESeq2 log_2_ fold change for SSpot relative to SGreen; red and blue denote higher and lower expression in SSpot, respectively. All transcripts met ∣log_2_FC∣ ≥ 1 and FDR < 0.05 in this contrast. C, abbreviated cluster identifier; OEC, oxygen-evolving complex.

SSpot contained less total chlorophyll than either green tissue (**Figure 2B**). Eighteen sequence-supported transcripts associated with chlorophyll turnover, chloroplast division, photosystem assembly, light harvesting or photosystem components met the differential-expression criteria in the SSpot–SGreen comparison (**Figure 5**). These comprised one chlorophyll-degradation candidate, one chloroplast-division factor, two photosystem-assembly factors, eight LHCII-associated transcripts, three PSI-associated transcripts and three PSII or oxygen-evolving-complex transcripts.

An SGR-like magnesium dechelatase transcript was higher in SSpot (log_2_FC = 1.13, FDR = 1.13 × 10^−^²¹). FtsZ2-1-like, PYG7-like and OHP2-like transcripts were also higher in SSpot. Four of the eight LHCII-associated transcripts were higher in SSpot and four were lower. LHCB7-like, LHCII-I CAB C1718.11, LHCB6/CP24-like C32742.1 and partial LHCII-I CAB C30366.26 were higher, whereas LHCB4.1/CP29.1-like C21239.0, LHCB6/CP24-like C4326.0, LHCB2-like C30366.17 and partial LHCII-I CAB C30366.20 were lower. Among the PSI-associated transcripts, PsaL-like C31482.0 and PsaE-like C23560.0 were higher, whereas PsaL-like C14601.0 was lower. Within the PSII and oxygen-evolving complex group, PsbY-like C13255.0 and PsbO/OEE1-like C33102.0 were higher, whereas PsbA/D1 C31141.0 was lower. The antenna and photosystem components therefore differed in both direction and magnitude. Transcripts annotated as HEMA, CHLG, CAO and POR were also detected, but none met the DEG criteria in the SSpot–SGreen comparison.

### 3.6 Anthocyanin-related and TF-family candidates in the local SSpot–SGreen contrast

**Figure 6:**
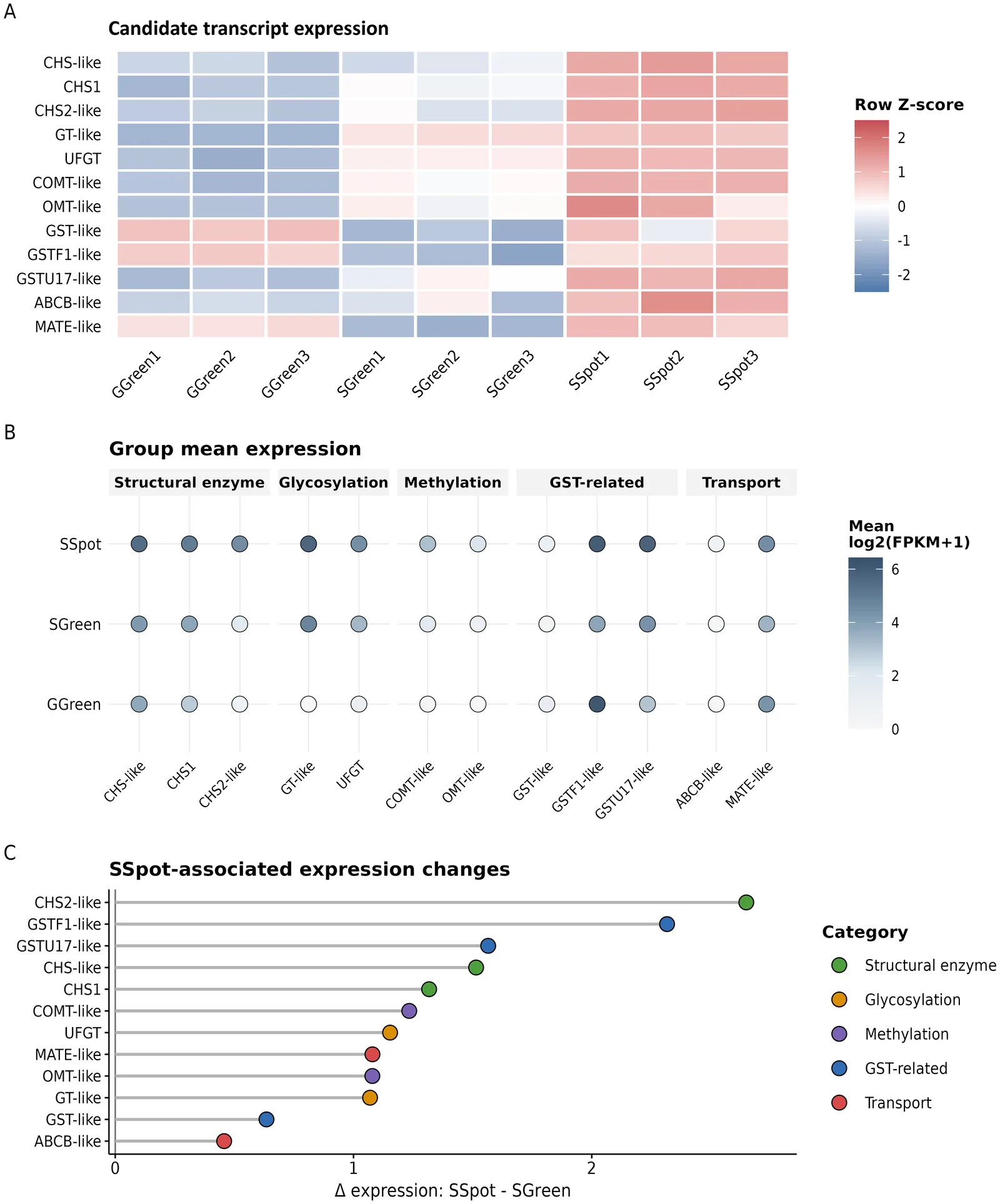
Expression profiles of selected anthocyanin-related candidate transcripts. **(A)** Row-scaled expression of selected candidates across GGreen, SGreen and SSpot. Each column represents one independently processed spatial subsample. **(B)** Mean expression of candidates assigned to putative biosynthetic, glycosylation, methylation, GST-related and transport-associated categories. **(C)** Mean expression differences between SSpot and adjacent SGreen, calculated from log_2_(FPKM + 1)-transformed values. Positive values indicate higher expression in SSpot. The 12 candidates were selected from SSpot–SGreen DEGs on the basis of annotations supporting putative roles in anthocyanin biosynthesis, modification or transport. Candidates designated “-like” were treated as sequence-similarity-based candidates rather than functionally validated enzymes or transporters.

Twelve transcripts annotated in anthocyanin biosynthesis, modification or transport were selected from the SSpot–SGreen comparison (**Figure 6**). These included three CHS-associated transcripts, two glycosylation-related candidates (GT-like and UFGT), two methylation-related candidates (COMT-like and OMT-like), three GST-associated transcripts, and the putative transporters ABCB-like and MATE-like.

All 12 selected candidates were more highly expressed in SSpot than in adjacent SGreen, although the magnitude of the difference varied considerably among transcripts. CHS2-like showed the largest increase, followed by GSTF1-like, GSTU17-like, CHS-like and CHS1. Smaller differences were observed for COMT-like, UFGT, MATE-like, OMT-like, GT-like, GST-like and ABCB-like. The CHS- and GST-associated candidates showed the clearest sector differences in the expression heatmap, while several modification- and transport-associated transcripts differed more modestly between the two sectors. Other flavonoid-pathway genes did not show the same SSpot-biased pattern. The canonical CHI transcript was detected but was not differentially expressed between SSpot and SGreen. One transcript annotated as F3′H (Cluster-32267.5) met the DEG criteria and was less abundant in SSpot than in SGreen (log_2_FC = −1.08, FDR = 0.0084). Other F3′H-related transcripts did not meet the predefined DEG threshold. An F3′5′H-like transcript and several DFR-related transcripts were also detected, but none met the DEG criteria. No transcript could be assigned unambiguously to canonical ANS/LDOX. A separate CHI-like DEG showed inconsistent annotations across databases and was therefore not assigned as CHI. Two transcripts annotated as anthocyanin malonyltransferase-like were detected, but neither met the predefined DEG criteria. Several BAHD- or SCPL-related acyltransferases were differentially expressed, although their annotations did not support their assignment as anthocyanin-specific acyltransferases.

**Figure 7:**
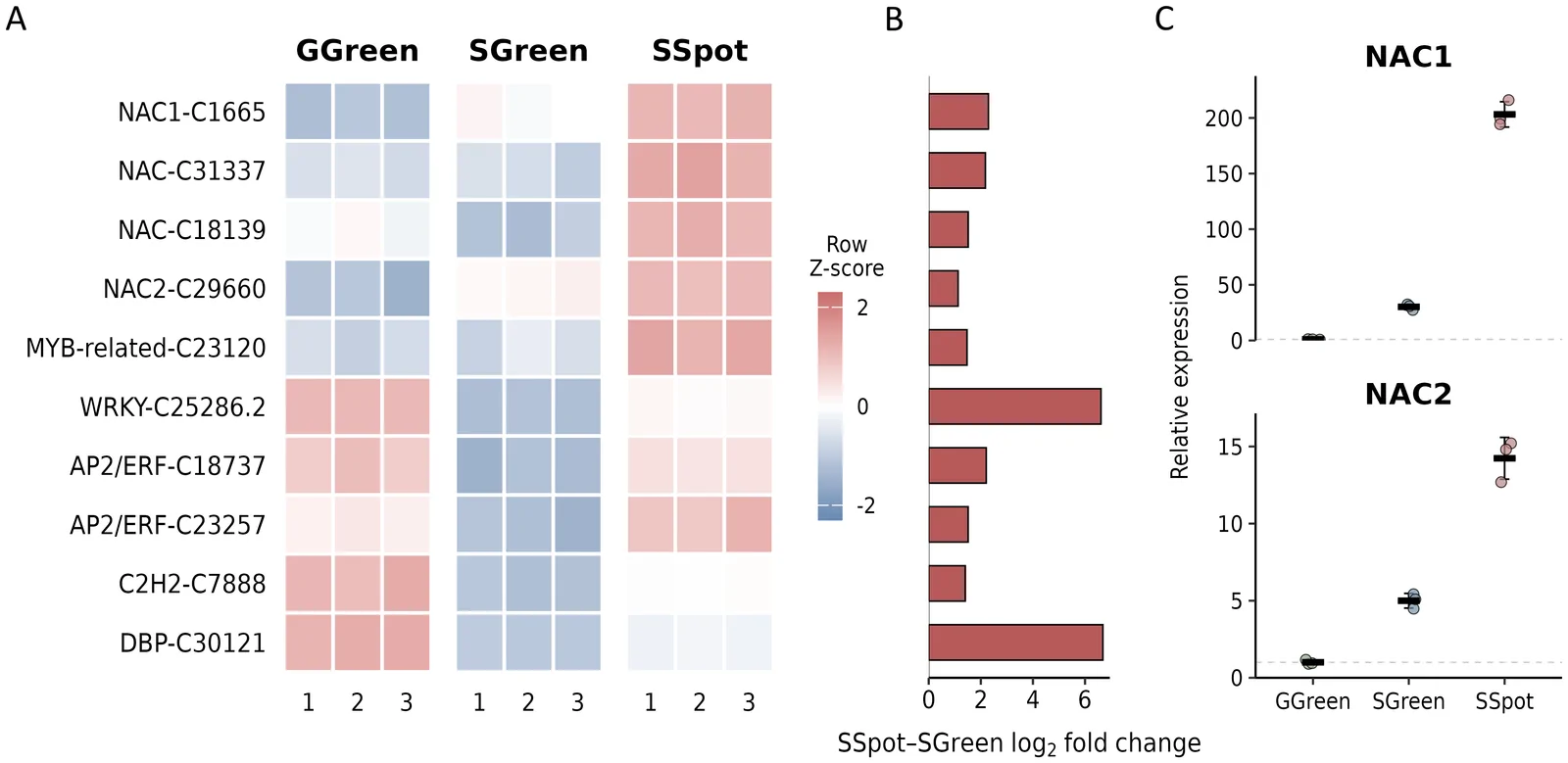
Expression profiles of representative TF-family candidate transcripts and qRT–PCR measurements. **(A)** Row-standardized log_2_(FPKM + 1) expression of 10 representative candidates across three spatial subsamples of GGreen, SGreen and SSpot. Numbers 1–3 indicate the spatial subsamples, and colour denotes the row Z-score. **(B)** DESeq2 log_2_ fold changes for the same transcripts in the SSpot–SGreen comparison. **(C)** Relative expression of NAC1 and NAC2 measured by qRT–PCR using one pooled sample per tissue type. Points represent three technical PCR reactions, with mean ± s.d. shown for each pooled sample. Technical reactions were not treated as independent biological replicates or used for inferential statistical testing. Y-axis ranges are independent for NAC1 and NAC2.

Seventy-one TF-family transcripts were differentially expressed between SSpot and SGreen (**Supplementary Figure 2**). Ten were selected for the representative panel: NAC-C1665, NAC-C31337, NAC-C18139, NAC-C29660, MYB-related-C23120, WRKY-C25286.2, AP2/ERF-C18737, AP2/ERF-C23257, C2H2-C7888 and DBP-C30121 (**Figure 7**). All ten had positive DESeq2 log_2_ fold changes in SSpot relative to SGreen, with the largest differences observed for WRKY-C25286.2 and DBP-C30121 (**Figure 7B**). Their expression across the three tissue types was more variable. The NAC and MYB-related transcripts were most abundant in SSpot, whereas WRKY-C25286.2, C2H2-C7888, DBP-C30121 and the two AP2/ERF candidates were also relatively abundant in GGreen but lower in SGreen before increasing again in SSpot (**Figure 7A**). NAC-C29737 showed a similar SSpot-biased profile among the five differentially expressed NAC-family transcripts (**Supplementary Figure 3**).

qRT–PCR results from the pooled samples were compared with the RNA-seq profiles for selected TF-family candidates. NAC1 and NAC2 were the only qRT–PCR targets included in the principal TF panel, and both were more highly expressed in SSpot than in SGreen in both datasets (**Figure 7C**). Across all 15 qRT–PCR targets, 14 were more highly expressed in SSpot than in SGreen, while FAR1 showed the opposite pattern (**Supplementary Figure 4**). Of the 14 targets with matching SSpot–SGreen DESeq2 estimates, nine changed in the same direction and five in the opposite direction (**Supplementary Table 1**). Data from the individual technical PCR reactions are provided in **Supplementary Data 3**.

### 3.7 Candidate transcript abundance covaries with anthocyanin composition across sampled leaf sectors

**Figure 8:**
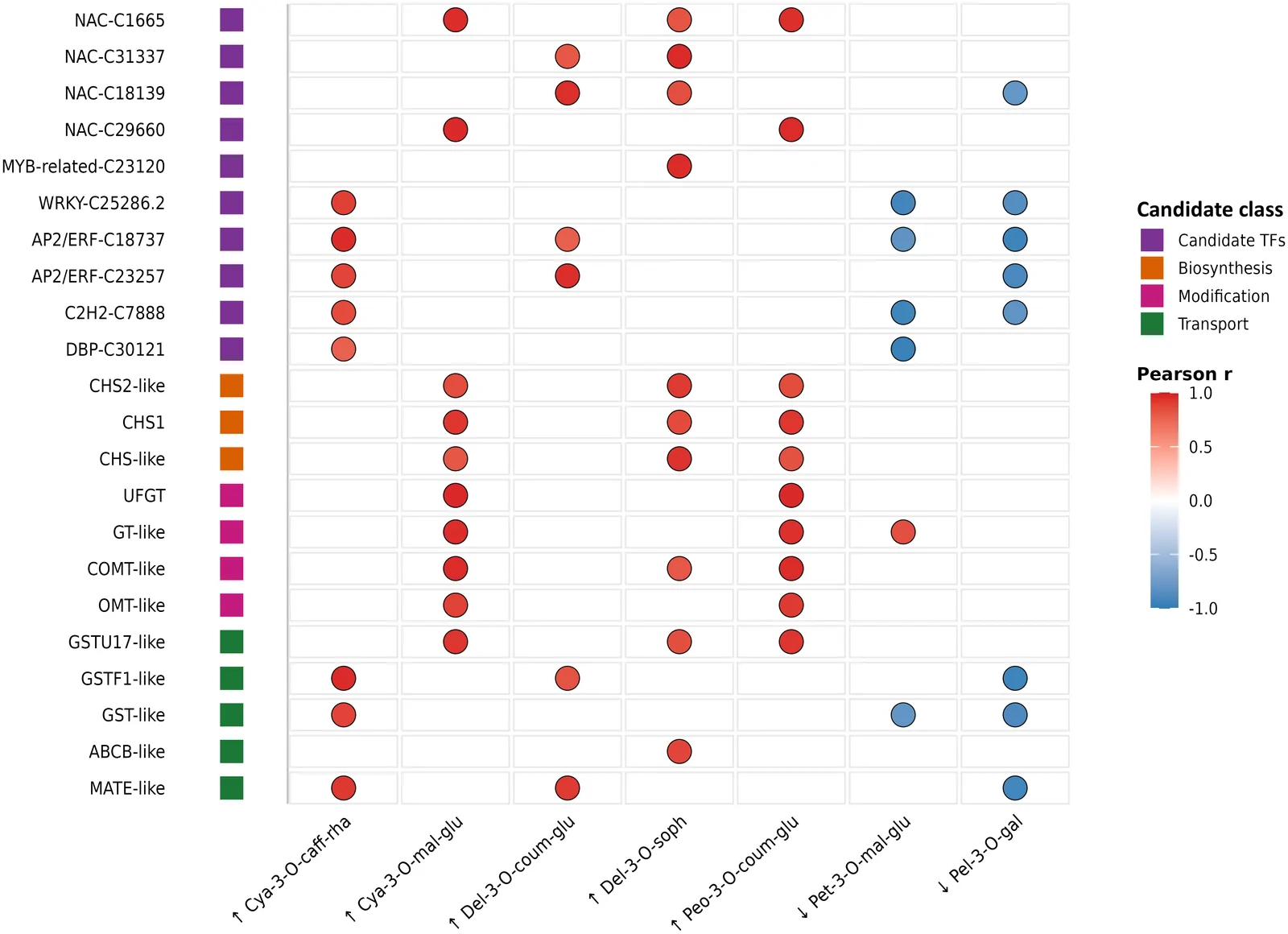
Covariation between candidate transcripts and anthocyanins across sampled leaf sectors. Pearson correlations were calculated across the nine spatial subsamples using log_2_(FPKM + 1)-transformed transcript abundance and log_2_-transformed metabolite abundance. The matrix includes 10 representative TF-family candidates, 12 candidates annotated in anthocyanin biosynthesis, modification or transport, and the seven fold-change-selected anthocyanins that differed between SSpot and SGreen. The coloured strip denotes candidate class. Only correlations with ∣r∣ ≥ 0.80 and Benjamini–Hochberg FDR < 0.05 are shown; circle colour indicates the sign and magnitude of Pearson’s r. Upward and downward arrows denote higher and lower anthocyanin abundance in SSpot relative to SGreen, respectively. Blank cells did not meet these thresholds.

Across the nine spatial subsamples, 58 of the 154 candidate transcript–anthocyanin pairs (37.7%) had ∣r∣ ≥ 0.80 and a Benjamini–Hochberg FDR < 0.05 (**Figure 8**). Associations occurred in each of the four candidate classes. All 44 associations involving the five anthocyanins enriched in SSpot were positive. Cyanidin-3-O-(6-O-malonyl-*β*-D-glucoside), delphinidin-3-O-sophoroside and peonidin-3-O-(6-O-*p*-coumaroyl)-glucoside each correlated with ten candidate transcripts. Cyanidin-3-O-(6″-O-caffeoyl)rhamnoside and delphinidin-3-O-(6-O-*p*-coumaroyl)-glucoside correlated with eight and six transcripts, respectively.

The two anthocyanins depleted in SSpot showed predominantly negative relationships. Pelargonidin-3-O-galactoside was negatively correlated with eight candidates. Petunidin-3-O-(6-O-malonyl-*β*-D-glucoside) was negatively correlated with five candidates but positively correlated with GT-like. Thus, the 58 significant pairs comprised 44 positive associations with SSpot-enriched anthocyanins and 14 associations involving the two depleted derivatives, 13 of which were negative.

## 4 Discussion

### 4.1 Pigment balance defines the local colour contrast

SSpot differed from adjacent SGreen primarily in chlorophyll content and anthocyanin composition, not in the total abundance of detected anthocyanins. Although the summed anthocyanin pool was similar in the two sectors, cyanidin- and peonidin-derived compounds were more prominent in SSpot, while petunidin- and pelargonidin-derived compounds contributed more strongly to the SGreen profile. The local colour contrast reflected a change in pigment balance rather than a general increase in anthocyanin abundance. A comparable relationship has been reported in *Banksia* floral styles, where colour variation followed anthocyanin and flavonol composition more closely than total pigment content (Stimpson et al., 2021). Despite the anatomical differences between flowers and leaves, both cases illustrate how pooled pigment measurements can conceal chemically distinct colour states.

At the level of individual compounds, only seven anthocyanin derivatives met the predefined fold-change threshold in the SSpot–SGreen comparison, with five increasing and two decreasing in SSpot. Several carried malonyl, caffeoyl or coumaroyl substituents, groups known to influence anthocyanin stability and spectral properties. Anthocyanin acylation also contributes to flower colour in grape hyacinth (Cao et al., 2024). In *T. macropoda*, these substituted derivatives form part of the chemical distinction between neighbouring sectors, although their individual contributions to the observed colour were not measured.

The pronounced reduction in chlorophyll may have increased the visual contribution of non-green pigments by weakening the green background (Bhardwaj et al., 2025; Wu et al., 2024; Zhu et al., 2024). Anthocyanin colour in intact tissue is also influenced by vacuolar pH, metal complexation, co-pigmentation, tissue anatomy and pigment localization (Trouillas et al., 2016; Yoshida et al., 2009). The measured anthocyanins alone do not fully account for the brown appearance of SSpot. Chlorophyll breakdown products, oxidized or polymerized phenolic compounds and pigments of fungal origin were not measured in this study, so their contribution to the observed colour was not assessed. SSpot is characterized here by reduced chlorophyll and an altered anthocyanin profile, without assigning its brown coloration to a single pigment class or process.

### 4.2 Adjacent green tissue provides a within-leaf reference for the dark sector

Most transcriptomic variation separated GGreen from the two sectors of the spotted leaf. The broad overlap and near-complete directional agreement between the two GGreen-referenced DEG sets showed that much of the expression variation was shared by SGreen and SSpot rather than confined to the dark sector. The SSpot–SGreen contrast contained fewer DEGs than either GGreen-referenced contrast. A comparable pattern has been reported in *Heliopsis helianthoides*, where contrasting leaf sectors retained broad molecular similarities alongside localized differences in metabolite and transcript profiles (Qin et al., 2024).

GGreen was sampled from an upper non-spotted leaf, whereas SGreen and SSpot were obtained from a lower spotted leaf. Comparisons involving GGreen also capture differences in leaf identity and position, with a possible contribution from developmental age. Because developmental status was not assessed independently, age-related variation cannot be disentangled from other between-leaf differences. SGreen and SSpot were sampled from adjacent regions of the same lower leaf, providing a more direct comparison between the dark sector and its immediate green background. This same-leaf contrast forms the principal basis for interpreting the chloroplast- and anthocyanin-associated candidates discussed below.

### 4.3 Local transcriptional features associated with the dark sector

GO enrichment of the SSpot–SGreen DEGs included flavonoid biosynthetic and metabolic processes, phenylpropanoid biosynthesis and chalcone synthase activity. Seven genes from the same contrast mapped to KEGG flavonoid biosynthesis, and the pathway met the applied multiple-testing threshold. Considered alongside the small set of fold-change-selected anthocyanins, the enrichment profile is more consistent with a focused pigment-associated expression shift than with pathway-wide activation. Chlorophyll- and photosynthesis-related differences were represented by a separately curated set of transcripts associated with chlorophyll degradation, chloroplast division, photosystem assembly and individual photosystem components.

Within this set, an SGR-like magnesium dechelatase transcript was more abundant in SSpot. STAY-GREEN proteins participate in chlorophyll catabolism in other plants (Ren et al., 2023; Sato et al., 2007; Shimoda et al., 2016), consistent with the lower chlorophyll content measured in SSpot. Chloroplast-division and photosystem-assembly candidates were also more highly expressed in SSpot, but the antenna and photosystem components showed no common direction: LHCII-, PSI- and PSII-associated transcripts included both increases and decreases. Nor did any transcript annotated to chlorophyll biosynthesis or tetrapyrrole precursor metabolism meet the same differential-expression criteria. The chlorophyll difference between SSpot and SGreen was accompanied by changes in selected chloroplast-associated transcripts rather than a uniform decrease in photosynthesis-related expression (Wu et al., 2024).

The anthocyanin-related candidates showed a more consistent local bias. All 12 were more highly expressed in SSpot than in adjacent SGreen, with the largest differences among CHS- and GST-associated transcripts; candidates assigned to glycosylation, methylation and transport changed more modestly. These expression differences were not accompanied by a higher total abundance of detected anthocyanins in SSpot. Instead, SSpot and SGreen differed in the abundance of individual anthocyanin derivatives, indicating that the main metabolic difference between the two sectors lay in anthocyanin composition.

Studies in other species provide functional context for the GST- and transporter-like candidates. Anthocyanin-associated GSTs have been linked to sequestration, transport or accumulation in several species, including RsGSTF12 in radish (Niu et al., 2022), BrGSTF12 in zicaitai (Fu et al., 2025) and MrGST1 in Chinese bayberry (Xue et al., 2022). MATE transporters also participate in vacuolar flavonoid transport; in grapevine, AM1 and AM3 mediate the transport of acylated anthocyanins across the tonoplast (Gomez et al., 2009; Pucker and Selmar, 2022). In SSpot, the UFGT, COMT-like, OMT-like, GST-related, ABCB-like and MATE-like candidates were more highly expressed than in adjacent SGreen, coinciding with differences in individual anthocyanin derivatives despite similar total anthocyanin abundance. These observations are consistent with a possible contribution of anthocyanin modification and intracellular transport or sequestration to the local compositional shift, although the substrates and cellular functions of these candidates have not been determined in *T. macropoda*.

The TF candidates were less uniform across the three tissues. All ten representatives were more highly expressed in SSpot than in SGreen, yet their abundance in GGreen varied: NAC and MYB-related candidates were most prominent in SSpot, while several WRKY-, AP2/ERF-, C2H2- and DBP-family transcripts were also relatively abundant in GGreen. NAC-family proteins have been linked to chlorophyll degradation in other species, including regulation of SGR-associated senescence pathways, and individual NAC factors can also influence anthocyanin-related processes (Oda-Yamamizo et al., 2016; D’Incà et al., 2023). In SSpot, the NAC candidates with higher expression occurred alongside higher SGR-like expression and lower chlorophyll content. These parallel changes do not establish a regulatory relationship between the NAC and SGR-like candidates. Anthocyanin-associated transcription commonly involves combinations of TF families (Gonzalez et al., 2008; Li et al., 2022; Xia et al., 2024), and NAC and WRKY proteins have been linked to pigment phenotypes in grape and pear (Jin et al., 2025; Zhang et al., 2025). The specific regulatory roles of these TF candidates in *T. macropoda* remain unresolved.

### 4.4 Transcript–anthocyanin associations highlight a focused candidate set

The transcript–anthocyanin matrix highlighted a sector-aligned pattern among the selected candidates. All significant associations involving the five anthocyanins enriched in SSpot were positive, whereas the two depleted derivatives showed negative associations. The associated transcripts included both TF-family candidates and genes annotated in anthocyanin biosynthesis, modification and transport (He et al., 2025). The correlations were calculated across all nine spatial subsamples after both feature sets had been selected from the SSpot–SGreen contrast. They therefore capture coordinated variation among sector-associated features without establishing direct molecular interactions.

qRT–PCR provided an additional comparison for a subset of TF-family candidates. NAC1 and NAC2, the only qRT–PCR targets represented in the principal TF panel, showed the same SSpot-biased expression direction as the RNA-seq data. The broader qRT–PCR panel showed less consistent agreement with RNA-seq. These pooled measurements provide a descriptive comparison with the RNA-seq expression patterns and do not constitute independent biological validation.

### 4.5 Study scope and future validation

The sector-resolved design captured pigment and transcriptomic variation within a single naturally patterned *T. macropoda* individual, with SSpot and adjacent SGreen providing the most direct local comparison. The three independently processed samples within each tissue type represent spatial subsamples rather than independent plant-level biological replicates. The differences reported here therefore define spatial patterns within the sampled individual, while their recurrence across plants remains to be tested. Each sampled sector also contains multiple cell types, so the present data resolve differences at the tissue-sector level but not at the level of individual cell types (Bhardwaj et al., 2025; Qin et al., 2024; Yin et al., 2023). Replication across independent plants and leaves, together with higher-resolution spatial analyses, would allow the reproducibility and tissue distribution of the principal patterns identified here to be examined further.

## 5 Conclusions

Within the sampled *T. macropoda* individual, SSpot represented a local pigment and expression state distinct from both the adjacent green tissue and the non-spotted reference leaf. SSpot contained approximately half the chlorophyll measured in SGreen, but the two sectors did not differ significantly in the summed abundance of detected anthocyanin derivatives. Their visible contrast was instead associated with seven fold-change-selected anthocyanins and a focused redistribution among pigment classes. The transcriptome data distinguished leaf-context effects from the SSpot–SGreen contrast: most variation distinguished GGreen from both tissues of the spotted leaf, whereas 1,459 differentially expressed genes defined the SSpot–SGreen contrast. Chlorophyll-, anthocyanin- and TF-associated transcripts, together with their metabolite covariation, provide a prioritized candidate set rather than evidence of direct regulation. Replication across individuals and leaves, followed by spatial localization and functional testing, is required to determine whether these sector-associated features are recurrent and causal.

## Data Availability

The sequencing datasets generated and/or analysed during the current study are available in the Genome Sequence Archive (GSA) under project accession number PRJCA060754 (https://ngdc.cncb.ac.cn/gsa/). Other data supporting the findings of this study are included in the article and/or its **Supplementary Material**.
