# Supplementary material for: Chlorophyll depletion and anthocyanin compositional shifts distinguish spotted leaf sectors in Tricyrtis macropoda
Source: Front Plant Sci. 2026 Sep 10;17:1949134. doi: 10.3389/fpls.2026.1949134 (PMC13601236; doi:10.3389/fpls.2026.1949134)
Supplement: Supplementary file 1 [file SupplementaryFile1.docx]

**Supplementary Figure S1. RNA-seq sample similarity among the nine spatial subsamples.** Pearson correlation matrix based on log₂(FPKM + 1)-transformed expression values for all expressed genes.

**Supplementary Figure S2. Ranked expression changes of 71 differentially expressed transcription-factor-family transcripts in the SSpot–SGreen contrast.** Bars show DESeq2 log₂ fold changes for transcripts meeting the differential-expression criteria of |log₂ fold change| ≥ 1 and FDR < 0.05. Positive and negative values indicate higher and lower expression in SSpot relative to SGreen, respectively. Colours distinguish the four NAC-family transcripts included in Figure 7, the six other Figure 7 candidates, one additional differentially expressed NAC-family transcript and the remaining differentially expressed TF-family transcripts.

**Supplementary Figure S3. Expression profiles of differentially expressed NAC-family transcripts.** (A) Row-standardized log₂(FPKM + 1) expression values across three independently processed spatial subsamples of GGreen, SGreen and SSpot. Colours indicate relative expression within each transcript. (B) DESeq2 log₂ fold changes for SSpot relative to SGreen. Bars follow the transcript order shown in A. Positive values indicate higher expression in SSpot. Four transcripts were included in the representative panel in Figure 7, whereas NAC-C29737 was retained as an additional NAC-family candidate. C, abbreviated transcript assembly identifier.

**Supplementary Figure S4.** **qRT–PCR expression profiles of 15 transcription-factor-family assays.** One pooled sample was analysed for each tissue type (GGreen, SGreen and SSpot). Relative expression was calculated using the 2^−ΔΔCt method, with *atp1* as the reference gene and the GGreen pool as the calibrator. Points represent three technical PCR reactions from each pooled sample; horizontal lines and error bars denote their mean and s.d., respectively. The y axis is logarithmic. Technical reactions were not treated as independent biological replicates, and no inferential statistical tests were applied.

**Supplementary Table S1. qRT–PCR assay information, expression summaries and correspondence with RNA-sequencing transcripts.** The table provides the TF-family assignment, corresponding RNA-seq transcript identifier, forward and reverse primer sequences, amplicon size and qRT–PCR expression summary for each of the 15 target assays. RNA-seq mean FPKM values, SSpot–SGreen log₂ fold changes, FDR values, DEG status, directional concordance between qRT–PCR and RNA sequencing, and inclusion in the principal TF candidate panel are also shown. The *atp1* reference assay is listed separately. One pooled sample was analysed for each tissue type (GGreen, SGreen and SSpot). qRT–PCR values are reported as the mean ± s.d. of three technical PCR reactions from each pooled sample; these reactions were not treated as independent biological replicates. Directional concordance indicates agreement in the sign of the SSpot–SGreen change between qRT–PCR and RNA sequencing.

**Supplementary Table S2.** **Complete abundance profiles and class assignments of the 27 detected anthocyanin derivatives in GGreen, SGreen and SSpot.** The table provides sample-level abundance values for the nine independently processed spatial subsamples, mean abundance for each tissue type, aglycone-class assignments, and the corresponding SSpot–SGreen fold-change results. Anthocyanin contents are expressed as μg g⁻¹ DW.

**Supplementary Table S3. Differential-expression summary and mutually exclusive DEG-set intersections.** The table summarizes the numbers of genes with higher and lower expression in the SGreen–GGreen, SSpot–GGreen and SSpot–SGreen comparisons, together with the mutually exclusive intersections used to construct the UpSet plot in Figure 4A. DEGs were identified from raw read counts using DESeq2 and defined by |log₂ fold change| ≥ 1 and FDR < 0.05. Higher and lower expression are defined relative to the first-named tissue in each comparison. The table also reports the complete DEG union, the genes shared by the two GGreen-referenced comparisons, their directional agreement and the 263 genes assigned exclusively to the SSpot–SGreen DEG set under the applied thresholds.

**Supplementary Table S4. GO over-representation analysis of the SSpot–SGreen DEG set and the direction-consistent GGreen-referenced gene set.** GO over-representation analysis was performed separately for the complete set of 1,459 SSpot–SGreen DEGs and the 2,838 direction-consistent genes shared by the SGreen–GGreen and SSpot–GGreen comparisons. The table provides the GO identifier, term name, observed gene count, annotated gene-set size, background gene count, annotated background size, gene ratio, background ratio, fold enrichment, nominal P value, contributing transcript identifiers and Benjamini–Hochberg FDR. The GO-annotated background comprised 17,187 genes from the common tested universe.

**Supplementary Table S5. KEGG over-representation analysis of the SSpot–SGreen DEG set and the direction-consistent GGreen-referenced gene set.** KEGG over-representation analysis was performed separately for the complete set of 1,459 SSpot–SGreen DEGs and the 2,838 direction-consistent genes shared by the two GGreen-referenced comparisons. The table provides the KEGG pathway identifier, pathway name, observed gene count, annotated gene-set size, background gene count, annotated background size, gene ratio, background ratio, fold enrichment, nominal P value, contributing transcript identifiers and Benjamini–Hochberg FDR. The KEGG-annotated background comprised 6,846 genes from the common tested universe.

**Supplementary Data S1. Complete union of genes meeting the differential-expression criteria in at least one pairwise comparison.** The dataset contains the 6,158 unique genes meeting the DEG criteria in at least one of the SGreen–GGreen, SSpot–GGreen or SSpot–SGreen comparisons. For each gene, DEG-set membership, mutually exclusive intersection assignment, log₂ fold change and FDR are provided for all three comparisons. A value of 1 denotes membership in the corresponding DEG set, whereas 0 denotes failure to meet the applied DEG criteria in that comparison.

**Supplementary Data S2. Expression matrix and differential-expression statistics for the 263 genes assigned exclusively to the SSpot–SGreen DEG set.** The dataset provides sample-level FPKM values for the nine independently processed spatial subsamples, mean FPKM values for GGreen, SGreen and SSpot, and the DESeq2 log₂ fold change, FDR and expression direction for the SSpot–SGreen comparison. Assignment to this set denotes exclusive DEG-set membership under the applied thresholds and does not imply biological specificity to SSpot.

**Supplementary Data S3. Technical-reaction-level source data for Supplementary Figure S4.** The dataset provides the target assay, TF-family assignment, corresponding transcript identifier, pooled sample, technical-reaction number, target Ct, *atp1* Ct, ΔCt, ΔΔCt and relative expression calculated using the 2^−ΔΔCt method. Three technical PCR reactions are reported for each target and pooled sample. These reactions quantify technical variation within one pooled sample per tissue type and are not independent biological replicates.
